# Molecular divergence and genomic composition of B chromosomes in the fish *Cyphocharax modestus* (Characiformes, Curimatidae)

**DOI:** 10.1038/s41598-026-66448-7

**Published:** 2026-09-09

**Authors:** Natalia dos Santos, Francisco de Menezes Cavalcante Sassi, Rodrigo Zeni dos Santos, Caio Augusto Gomes Goes, Renata Luiza Rosa de Moraes, Fabio Porto-Foresti, Thomas Liehr, Marcelo de Bello Cioffi, Ricardo Utsunomia

**Affiliations:** 1 Faculdade de Ciências, Universidade Estadual Paulista, Bauru, SP 17033-360 Brazil; 2 Departamento de Genética e Evolução, Universidade Federal de São Carlos, São Carlos, SP 13565-905 Brazil; 3 Aquaculture Center of Unesp, São Paulo State University (Unesp), Jaboticabal - SP, 14884-900 Brazil; 4 Jena University Hospital, Friedrich Schiller University, Institute of Human Genetics, Jena, Germany

**Keywords:** Supernumerary chromosomes, satDNAs, Comparative genomic hybridization, Repetitive DNAs, Cytogenomic, Satellitome, Evolution, Genetics, Molecular biology, Zoology

## Abstract

B chromosomes are supernumerary elements that evolve from standard A chromosomes and are primarily composed of repetitive DNAs, yet their origin, diversification, and molecular composition remain poorly understood in most vertebrates. We investigated two allopatric populations of *Cyphocharax modestus* (Curimatidae) combining classical cytogenetics, comparative genomic hybridization (CGH), and comparative satellitomics to characterize the repetitive DNA landscape of its B chromosomes. While both populations exhibited a conserved karyotype of 2n=54 biarmed chromosomes, five individuals from the Batalha River (BR) carried supernumerary chromosomes, comprising two distinct variants: a C-positive B1 and an C-negative B2. Comparative satellitome analysis between 3B-carrying and B-lacking individuals identified 116 satellite DNAs (CmoSatDNAs), with the 3B library showing higher abundances of specific sequences. Fluorescence *in situ* hybridization (FISH) revealed that both B variants share two centromeric satellites (CmoSat01-192 and CmoSat02-108) with the A complement, while CmoSat58-47 was exclusively to B2. Minimum spanning tree analysis of CmoSat58-47 revealed B-exclusive haplotypes alongside haplotypes shared with B-lacking individuals, suggesting a recent origin for these chromosomes. CGH experiments further confirm the sequence sharing between the A and B chromosomes, supporting an intraspecific origin, and revealing substantial genomic differentiation among B variants.

**Supplementary Information:**

The online version contains supplementary material available at https://doi.org/10.1038/s41598-026-66448-7.

## Introduction

B chromosomes, also known as supernumerary or accessory chromosomes, are extra karyotypic units in addition to the A chromosomes found in all major eukaryotic taxa^1,2^. These chromosomes are an intriguing example of genomic conflict, as they are not essential to the normal life cycle of organisms and exhibit non-Mendelian inheritance^1,3^. Although a few studies suggest that B chromosomes originated interspecifically in some species^4–6^, the vast majority of occurrences indicate that these components originated intraspecifically as a result of chromosomal breakages, segmental duplications, centromere misdivision, unequal recombination, and whole-chromosome derivation followed by progressive divergence from the A complement^1,7–12^

The parasitic nature and evolutionary maintenance of B chromosomes are linked to higher transmission rates than those predicted by Mendelian rules, a phenomenon known as “drive”^2,13–16^. Once originated, the B chromosomes are believed to undergo a “life cycle”, starting with their invasion of the genome, followed by neutralization, and eventual loss, unless replaced by a novel B variant capable of re-establishing drive^1^. Indeed, the intraspecific diversification of B-variants has been widely reported in different groups, and they can show structural variations in morphology, size, C-heterochromatin amount, DNA content, and transmission^17–19^.

Transposable elements and tandemly repeated sequences (particularly satellite DNAs) are examples of repetitive elements that often play a significant role in the evolution of B chromosomes, which typically show limited or no recombination^1^. For this reason, several repetitive DNA classes have been observed in the B chromosomes of animals and plants, among which satellite DNAs (satDNAs) are one of the most prevalent ones^16,20–24^. These sequences are prone to accumulate in the B chromosomes, as a result of Muller’s ratchet-like mechanisms^1,25–27^. Consequently, the location of satDNAs on B or sex chromosomes can provide valuable insights into the evolutionary history of these elements, including their origin and subsequent differentiation.

*Cyphocharax modestus*^28^ popularly known as “saguirus”, is a Curimatidae fish distributed in South America and parts of Central America^29,30^ Curimatidae species exhibit a conserved diploid number 2n=54, with a karyotype mainly composed of biarmed chromosomes^31^, while some species/genera (e.g., *Cyphocharax* and *Steindachnerina*) also exhibit small supernumerary B chromosomes^31–35^. In *C. modestus*, the occurrence of B chromosomes was first reported by Venere and Galetti^36^. Indeed, B-carry populations of *C. modestus* were identified in the Tietê^36^, Piracicaba^37^, Tibagi^38^, and Paranapanema^33^ River Basins. Previous studies have documented intra- and interindividual variation in B chromosome number, ranging from one to four supernumerary chromosomes, as well as distinct B chromosome variants with differences in their heterochromatin content^32,36,38^. Furthermore, molecular cytogenetic analyses based on chromosome microdissection and chromosome painting have been applied in Curimatidae to investigate the origin and genomic composition of B chromosomes, revealing extensive sequence sharing between B chromosomes and the standard complement^34^. However, the reports so far have mostly focused on conventional descriptive cytogenetic data. In the present study, we analyzed two allopatric populations of *C. modestus* from CN (Campo Novo stream) and BR (Batalha River), integrating molecular cytogenetic and bioinformatic tools to unveil the composition and diversification of B chromosomes in this species. Using an integrated cytogenomic and satellitomic approach, we compared B-carrying and B-lacking individuals to assess sequence sharing with the A complement and molecular differentiation between coexisting B variants. We identified two B chromosome variants (B1 and B2) co-occurring within a single population that most likely originated intraspecifically and have since followed distinct evolutionary trajectories. This approach provides a framework for understanding the dynamics of accessory chromosome evolution in Curimatidae.

## Results

### Cytogenetic characterization

**Fig. 1:**
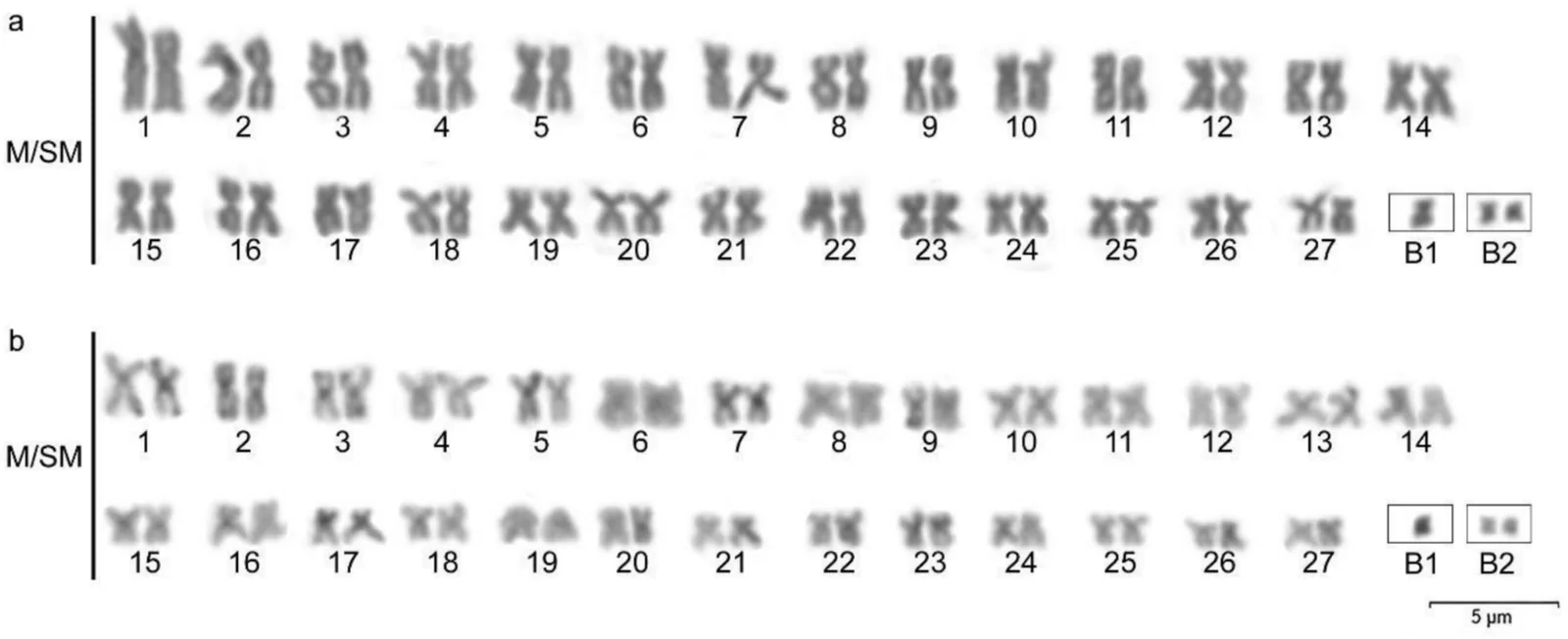
Karyotype of *C. modestus* (BR population) bearing three B chromosomes after conventional Giemsa staining (**a**) and C-banding (**b**). The box highlights the B_1_ and B_2_ variants, their respective morphologies, and their C-heterochromatin patterns. Scale Bar = 5 µm.

The analyzed specimens of *C. modestus* from both populations exhibited 2n=54 and a karyotype composed exclusively of biarmed chromosomes, without detectable sex-related polymorphisms **(**Fig. 1**).** In addition, five of the twelve individuals from the BR population carried small supernumerary chromosomes **(**Fig. 1**, **Table 1**):** three individuals harbored a single B chromosome, one carried three B chromosomes, and one carried five B chromosomes. C-banding revealed the presence of at least two B-chromosome variants in this population: (i) a small metacentric C-positive chromosome (hereafter B1) and (ii) an even smaller C-negative chromosome (hereafter B2) **(**Fig. 1**).** B chromosomes were not observed in any individuals from the CN population.

**Table 1:** Geographic coordinates of collection sites with the number and sex of specimens analyzed in the present study.

**Species**	**Collection sites**	**Population code**	**N**
Cyphocharax modestus	Campo Novo stream, Tietê River Basin 22°23’28.6"S 49°01’15.6"W	CN	(1♀; 1♂; 2 sex unidentified)
*Cyphocharax modestus*	Batalha River, Tietê River Basin 22°23’40.2"S 49°06’35.2"W	BR	(2♀; 5♂; 5 sex unidentified)

### Satellitome analysis

Independent analyses identified 105 satDNAs in the 0B individual and 74 satDNAs in the 3B individual. After merging the two catalogs and removing redundant sequences shared between the individuals, the final non-redundant species-level catalog comprised 116 satDNA families in *C. modestus* (Supplementary Table S1). These sequences were deposited in NCBI GenBank under accession numbers OR604368–OR604483. Homology analyses further grouped the satDNAs into 10 superfamilies (Supplementary Table S1).

Repeat unit lengths ranged from 16 to 2,520 bp, with a median of 252.6 bp. The catalog was predominantly composed of long satDNAs (>100 bp) and sequences with an A+T content above 50%. The median A+T content was 54.8%, and only 20 satDNAs had an A+T content below 50% (Supplementary Table S2). Overall, satDNA abundance patterns were similar between the 0B and 3B genomic libraries. The main exception was CmoSat58-47, which was approximately 34-fold more abundant in the 3B library than in the 0B library (Table 2).

**Table 2:** Main characteristics of the CmoSatDNAs amplified by PCR and used for FISH experiments.

**satDNA family**	**A+T (%)**	**Abundance 3B**	**Abundance 0B**	**Abundance 3B/0B**	**Divergence 0B (%)**	**Divergence 3B (%**
CmoSat01-192 CmoSat02-108 CmoSat03-74 CmoSat04-187 CmoSat06-317 CmoSat07-191 CmoSat09-772 CmoSat11-64 CmoSat31-66 CmoSat36-182 CmoSat37-43 CmoSat52-183 CmoSat53-56 CmoSat56-166 CmoSat58-47 CmoSat67-140	64.1 52.8 52.7 64.7 57.7 59.2 58.9 53.1 60.6 50.5 30.2 57.4 53.6 51.2 44.7 47.9	0.015 0.014 0.007 0.007 0.002 0.002 0.002 0.002 5.02E-04 3.72E-04 3.68E-04 2.43E-04 2.30E-04 2.08E-04 1.90E-04 1.53E-04	0.013 0.013 0.008 0.004 0.002 0.002 0.002 0.002 2.96E-04 1.74E-04 9.22E-04 4.40E-05 9.95E-05 5.64E-04 5.56E-06 6.80E-05	1.18 1.05 0.89 1.90 1.18 1.16 0.99 0.72 1.70 2.14 0.40 5.53 2.31 0.37 34.14 2.25	5.61 12.75 7.42 6.89 4.7 8.17 8.35 6.21 10.52 5.84 8.76 7.39 11.07 6.47 4.93 3.86	5.7 12.63 7.78 7.01 4.68 8.3 8.31 6.41 10.36 5.97 8.69 4.27 4.91 8.96 3.28 3.7

### Chromosomal distribution of CmoSatDNAs

**Fig. 2:**
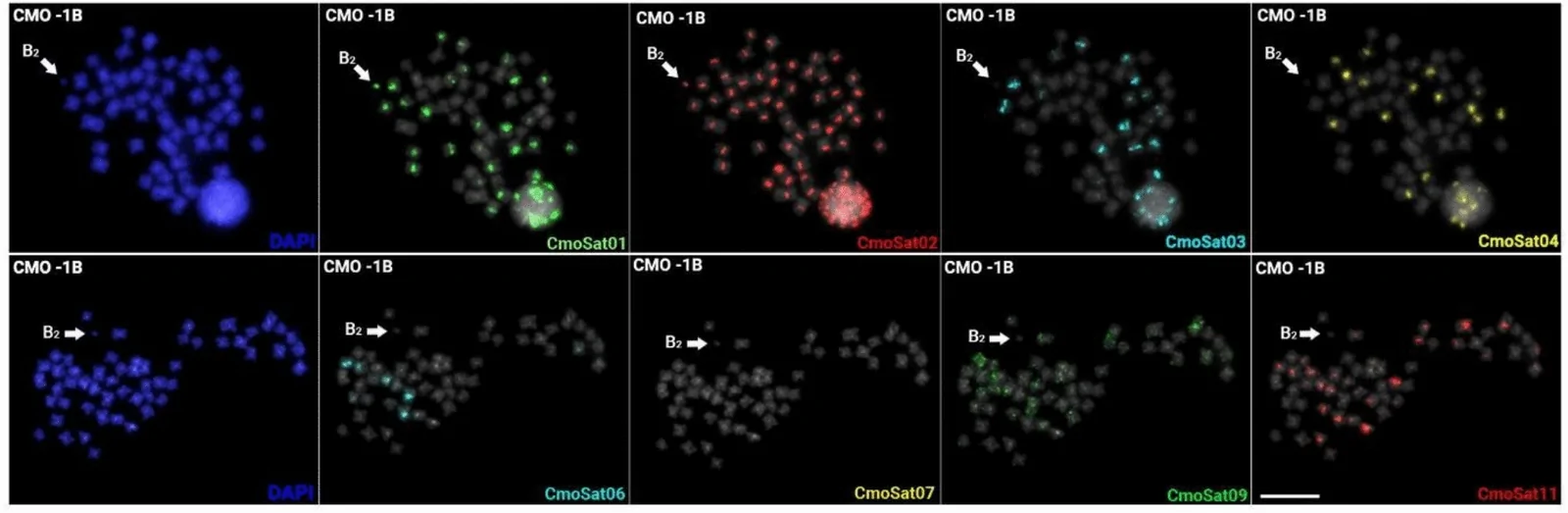
Metaphase plates of *Cyphocharax modestus* 1B_2_-carrying after FISH, highlighting the chromosomal location of CmoSatDNAs. The satDNA family names are indicated on the lower right, in red (Atto550-labeled), green (Atto488-labeled), light blue (Atto425-labeled), or yellow (Atto680-labeled). Arrows indicate the B chromosomes. Scale bar = 5µm.

**Fig. 3:**
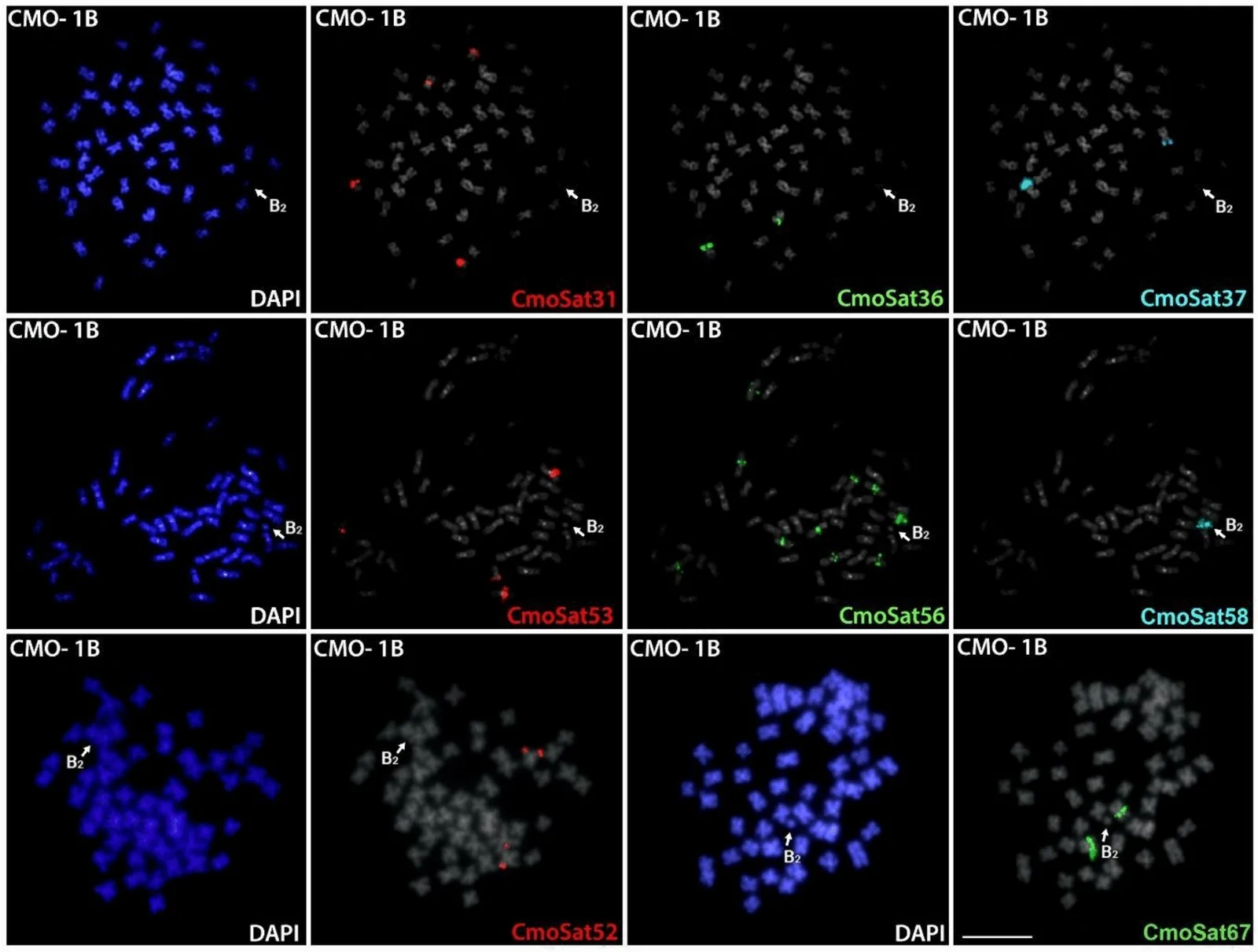
Metaphase plates from *Cyphocharax modestus* 1B_2_-carrying, highlighting the chromosomal location of satDNAs. The satDNA family names are indicated on the lower right, in red (Atto550-labeled), green (Atto488-labeled), or light blue (Atto425-labeled). The B chromosomes are indicated. Scale bar = 5µm.

**Fig. 4:**
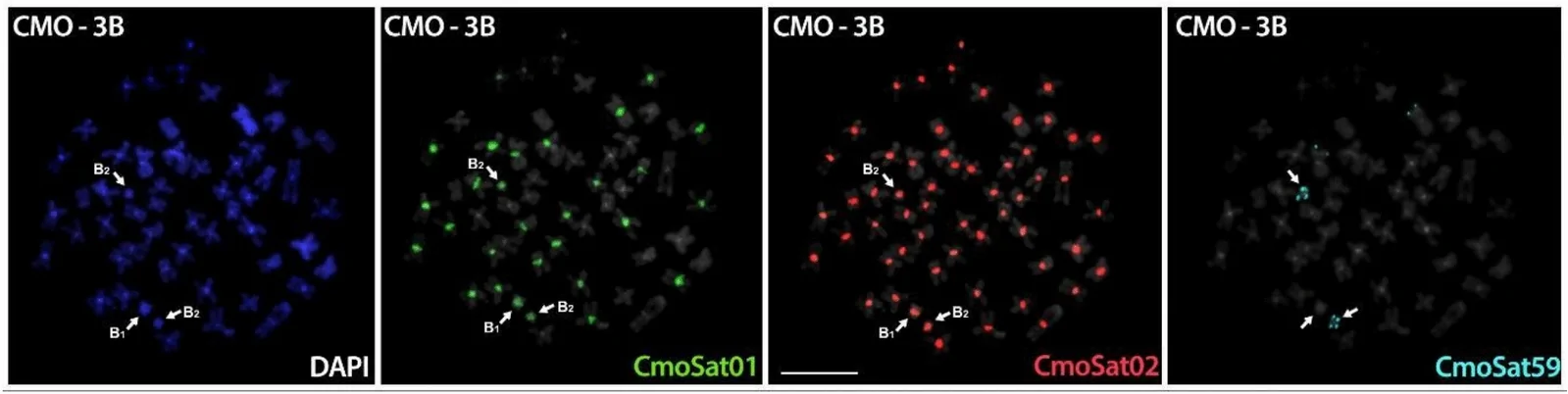
Metaphase from *Cyphocharax modestus* 3B_1,2_-carrying, highlighting the chromosomal location of the two most abundant and most differential satDNA that were mapped in the B chromosome of *C. modestus* - 1B_2_. The satDNA family names are indicated on the lower right in red (Atto550-labeled), green (Atto488-labeled), or light blue (Atto425-labeled). B chromosomes are indicated. Scale bar = 5 µm.

Fifteen out of the 22 selected CmoSatDNAs **(**Table 2**)** were successfully amplified by PCR and *in situ* mapped with FISH experiments. Individuals from both the BR and CN populations exhibited similar patterns of satellite DNA distribution on the A chromosomes, with most CmoSatDNAs localized to pericentromeric or telomeric regions. In contrast, CmoSat07-191 and CmoSat58-47 did not display clustered chromosomal localization (Fig. 2, 3; Supplementary Figure S1, S2). In specimens from the BR population, both B1 and B2 exhibited signals for CmoSat01-192 and CmoSat02-108, while CmoSat58-47 was exclusively mapped to B2 (Fig. 2, 3, and 4). This last satDNA also presents an additional signal to the autosomal pair carrying the 18S rDNA (Supplementary Figure S3) in 1B carrier individuals. Notably, the 5B individual showed signals for CmoSat58-47 that were not coincident with the 18S rDNA-carrier pair, revealing five copies of B2 (Supplementary Figure S4).

### Intra- and Intergenomic divergence

Patterns of intra- and intergenomic diversity for CmoSat58-47 were consistent with the abundance estimates obtained from RepeatMasker and FISH, with the 3B genomic library yielding approximately tenfold more monomers and fivefold more haplotypes than the 0B library **(**Table 3**).** A higher haplotype diversity was also observed in the 3B individual, likely due to diversification of monomers within the B chromosome. Notably, however, the four most abundant haplotypes were shared between the 0B and 3B libraries (Fig. 5), suggesting a relatively recent origin of B2.

**Table 3:** Genetic variation of CmoSat58-47 obtained from raw reads.

**satDNA**	**Sample**	**N**	**Hap**	**Hd**	**π**
CmoSat58-47	0B	99	19	0.878 ± 0.022	0.045
	3B	974	93	0.931 ± 0.005	0.053
	Total	1073	98	0.934 ± 0.004	0.053

N = number of sequences, Hap = number of haplotypes, Hd = haplotype diversity, π = nucleotide diversity

**Figu. 5:**
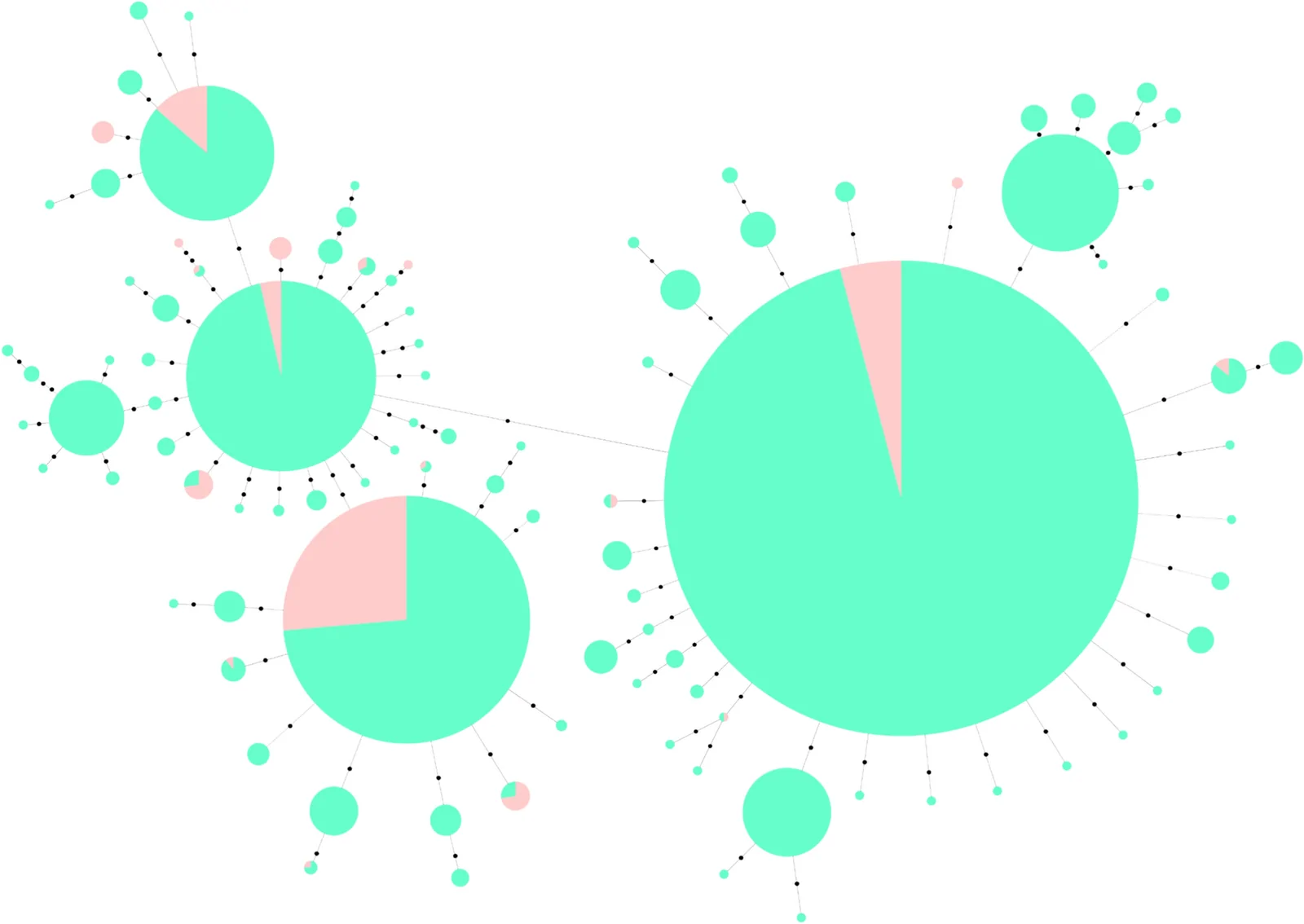
Linear MSTs of CmoSat58-47, obtained from reads of B-lacking (red) and 3B-carrying (green). The diameter of the circle is proportional to the abundance of the haplotype, the numbers represent the number of mutational steps, and black circles represent the 01 base pair of divergence between haplotypes.

### Comparative Genomic Hybridization (CGH)

**Fig. 6:**
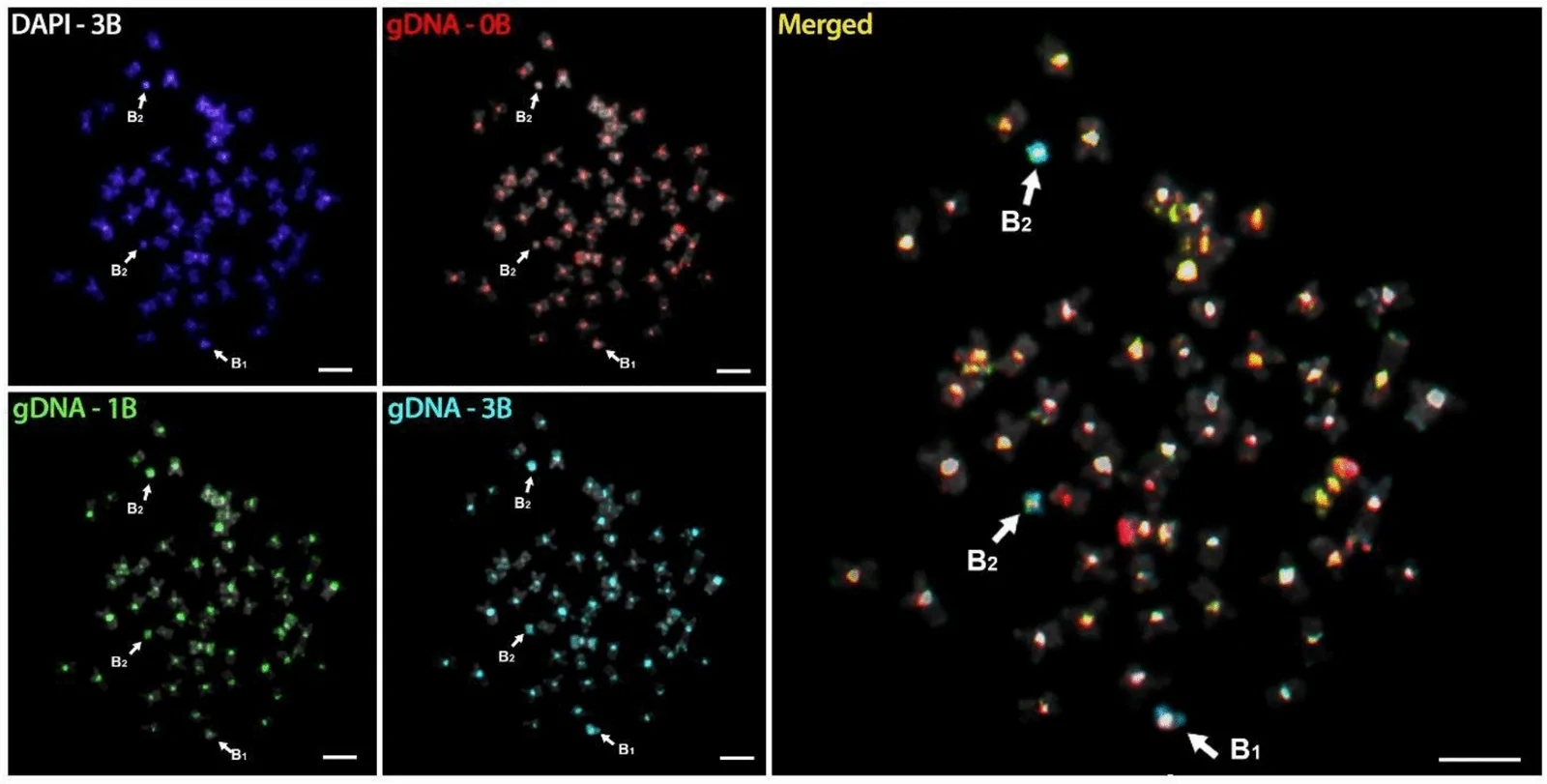
Mitotic metaphase from *Cyphocharax modestus* 3B_1,2_-carrying individual after interspecific comparative genomic hybridization (CGH). Chromosome spreads were probed with a 1B_2_-carrying (green), 3B_1,2_-carrying (light blue), and B-lacking (red) -derived genomic probes. The merged image contains all three merged genomic probes and DAPI counterstaining. The arrows indicate the B chromosomes. Scale bar = 10 µm

Comparative genomic hybridization (CGH) analyses comparing B-lacking individuals with those carrying either a single B2 chromosome (1B2) or both B1 and B2 variants (3B1,2) demonstrated extensive DNA compartmentalization, with a variety of non-overlapping variant-specific signals (Fig. 6). B-lacking individuals from the CN population showed more pronounced variant-specific hybridization patterns than the others from the BR population, potentially reflecting population-specific genomic rearrangements. With respect to the B chromosomes, the B-lacking probe hybridized to the centromeric regions of both B1 and B2, likely corresponding to the regions occupied by CmoSat01-192 and CmoSat02-108.

## Discussion

Accessory chromosomes are enigmatic elements that have been intensively investigated using both classical and molecular cytogenetic techniques to shed light on their origin and evolution. Here, we demonstrated the occurrence of two B-variants (B1 and B2) within a single population of the fish *C. modestus*, most likely arising intraspecifically. They both share two satDNAs that are present in several (i.e., CmoSat01-192), or all (i.e., CmoSat02-108) centromeres of the A complement. In monocentric species, some satDNAs can be associated with centromeres and play an important role in chromosome segregation during the cell cycle^39,40^, suggesting these sequences may be functionally important in B chromosomes as well. Despite sharing centromeric sequences, the two B variants appear to follow divergent evolutionary pathways, leading to differentiation in size (large B1 vs. small B2), C-heterochromatin patterns, and repetitive sequence accumulation (Fig. 1-4, 6). This diversification of variants has been previously observed in other *C. modestus* populations^33^, although the earlier study did not explore these variants in detail.

To investigate the molecular basis of these distinct evolutionary pathways, we described and compared the satellitomes of a 3B-carrying and a B-lacking (0B) individual. Although all CmoSatDNA families were detected in both sequencing libraries, several exhibited higher relative abundances in the 3B-carrying individual (Supplementary Table S1). Most notably, CmoSat58-47 was the only one mapped exclusively to the B2-variant, and was consistently detected in all individuals carrying one, three, or five B chromosomes **(**Fig. 3 and 4, Supplementary Figure S3). MST (Minimum Spanning Tree) analysis of CmoSat58-47 revealed B-exclusive haplotypes, although these are not among the most abundant variants **(**Fig. 5**).** The origin of several B-exclusive haplotypes that resulted from this satDNA amplification in the B chromosomes could have been triggered by preferential transposition to B, as already observed in grasshoppers^41,42^, or by errors in DNA repair mechanisms that can result in the formation of additional copies of repetitive elements^43,44^. Furthermore, the sequences shared between the A and B chromosomes may be related to the origin of these accessory elements, as they might constitute an intricate mosaic of independent duplicated segments of genetic material^45^. Notably, all CmoSatDNAs found in the B chromosomes of *C. modestus* had a large fraction of A+T nucleotides. Although the significance of this compositional bias remains debated, studies suggest that a high proportion of A+T helps in the DNA bending required to form heterochromatic regions^46^.

By investigating the chromosomal distribution of such CmoSatDNAs through FISH, we demonstrated that only three satDNAs are present on the B chromosomes of *C. modestus*: the centromeric CmoSat01-192 and CmoSat02-108, which occur in both variants, and the CmoSat58-47 which is exclusive to the C-negative B2 variant **(**Figs. 3, 4, and Supplementary Figure S4). Usually, the presence of B chromosomes does not substantially alter the overall heterochromatin content of the genome, suggesting the existence of a compensatory mechanism between A and B chromosomes^47,48^. The heterochromatin distribution in the *C. modestus* specimens herein investigated is restricted to the centromeric region of a few chromosomal pairs, in contrast to other populations of the same species that show strong C-bands in some telomeres and all centromeres (e.g.^49^. It is generally accepted that satellite DNAs may alter the nuclear structure and the physical properties of heterochromatin, both of which are critical for safeguarding proper chromosomal segregation^50^. Therefore, since both CmoSat01-192 and CmoSat02-108 were mapped at the centromeric region of most chromosomes, including those from the B complement **(**Fig. 2 and 4**),** it is possible that they are somehow related to regulatory function or act as major components of their centromeres. On the other hand, the lack of B-specific satDNAs for the B1 C-positive variant here observed might suggest that these B chromosomes had a recent origin due to their low level of differentiation^51^, since previous investigations on satDNA distribution on B chromosomes have shown that heterochromatic B chromosomes usually have a distribution of distinct repeated sequences (TEs, satDNAs), although they are usually enriched in satellite sequences, including some B-specific ones^8,52,53^.

Since the differences of B chromosomes in *C. modestus* are not restricted to the presence of variants but also on the population pool of these accessory elements, we compared the genomes of a 1B2-carrying, a 3B1,2-carrying, and a B-lacking individual through CGH experiments to gain insights into the total genomic similarities/differences among them. The high abundance of highly repeated DNA sequences (not only CmoSatDNAs) shared between the standard (A complement) and B chromosomes can be taken as supportive of the intraspecific origin of these B chromosomes **(**Fig. 6**).** To date, most B-chromosomal records indicate an intraspecific origin for these elements^1,22,54–59^. Shape variations in multiple B chromosomes stand out in different Characiformes species, such as *Astyanax scabripinnis*^60,61^, *Moenkhausia* species^62,63^, and *Prochilodus lineatus*^64^, showing that this scenario is not exclusive to *Cyphocharax* and might reflect a bigger picture yet to be explored.

Within the family Curimatidae, two main hypotheses have been proposed for the origin of B chromosomes: (i) multiple independent and relatively recent origins, and (ii) a single origin in the common ancestor of the family, followed by lineage-specific losses^31,38^. B chromosomes often exhibit mitotic and meiotic instability due to non-Mendelian behavior^34^, and their population incidence likely depends on interactions among natural selection, historical factors, transmission dynamics, and stochastic events^1^. The presence of only single B-specific satellite DNA family (CmoSat58-47) together with the sharing of haplotypes revealed by MST analysis **(**Fig. 5**)** supports a recent origin of these accessory chromosomes and may account for the limited satDNA differentiation observed between 0B and 3B individuals. Future comparative studies across populations of *C. modestus* from different river basins will be necessary to clarify whether B chromosomes share a common origin or arose independently in this species, thereby providing further insight into the evolutionary dynamics of accessory elements in Curimatidae.

## Conclusions

In this study, we characterized the repetitive DNA landscape of B chromosomes in *C. modestus,* revealing a complex scenario of shared ancestry and divergent evolution. Our findings confirm the presence of two distinct B variants (B1 and B2) in the BR population, suggesting an intraspecific origin, as the majority of their repetitive DNAs are shared with the A complement. The identification of a B2-exclusive satellite DNA (CmoSat58-47) and the presence of B-exclusive haplotypes demonstrate that these elements are not merely inert copies of A chromosome sequences but are capable of independent molecular evolution. The contrasting repetitive DNA profiles of the C-positive B1 and the C-negative B2 further suggest that these variants are following distinct evolutionary trajectories, potentially reflecting differences in their age, origin, or mechanisms of accumulation. Future research should therefore expand the geographic and populational sampling to test the "single versus multiple origin" hypotheses for B chromosomes in Curimatidae. Integrated genomic, transcriptomic, and epigenetic analyses are essential to determine whether B chromosome sequences are transcriptionally active, to characterize their centromere-specific histone variants, and to elucidate the molecular mechanisms driving their accumulation, transmission, and phenotypic effects.

## Material and methods

### Specimens and karyotype analyses

**Fig. 7:**
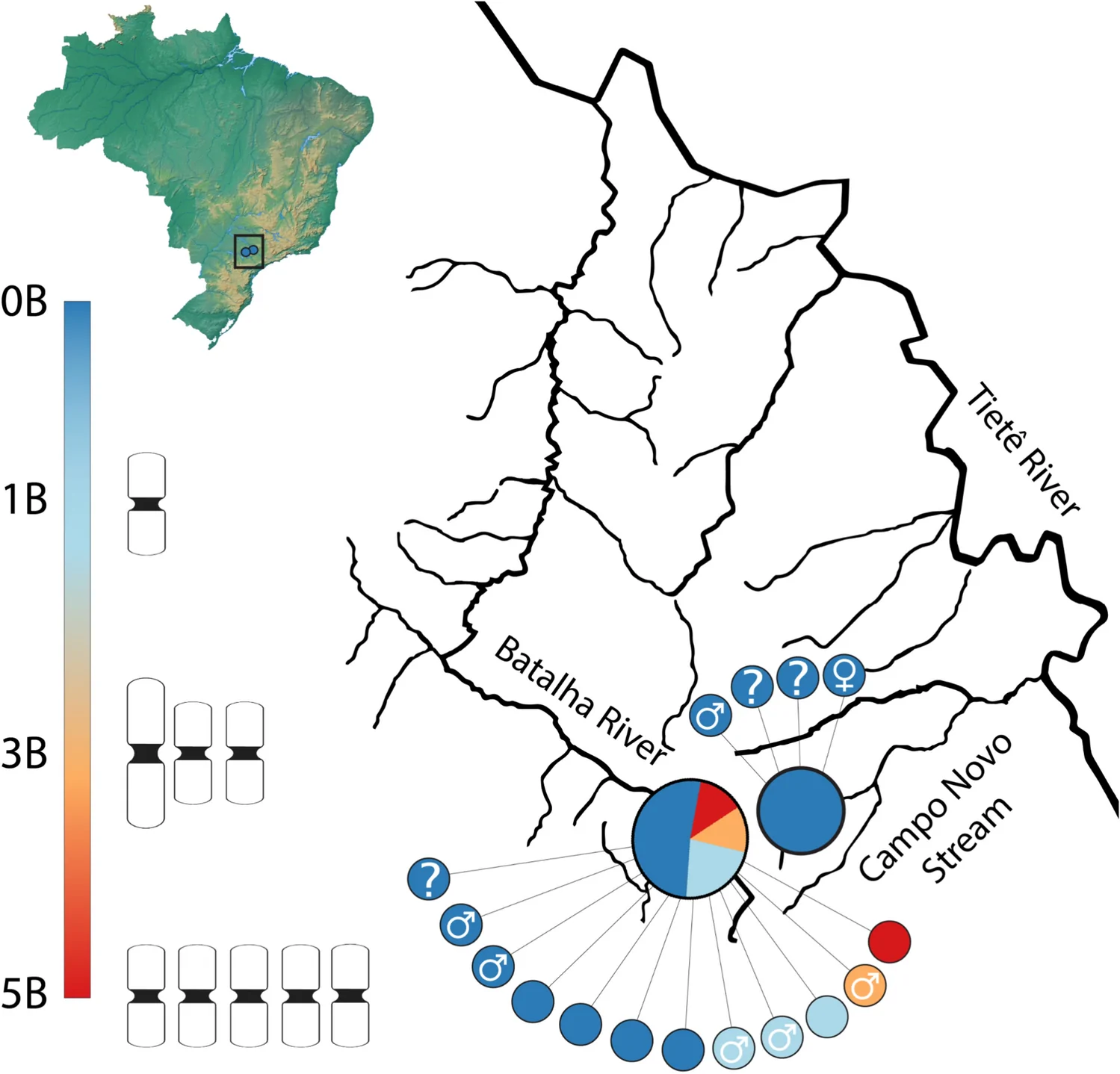
Map highlighting the collection sites and distribution of B-carrying individuals. Circles indicate the sample points (BR and CN in blue) in the Brazil map and in the approximated view of São Paulo State. Smaller circles in the closer view of the left bank of the Tietê River Basin represent each individual collected, as shown in Table 1. The color gradient indicates the number and variety of B chromosomes per cell in each individual investigated. Scale bar = 10km.

Two populations of *C. modestus* were analyzed: CN (Campo Novo stream) and BR (Batalha River), as specified in Table 1 and Fig. 7, with approval from the Brazilian environmental agencies ICMBio/SISBIO (License 3245). Firstly, cell suspensions containing mitotic metaphase chromosomes were obtained from anterior kidney cells following the protocol described by^65^. C-banding was performed according to^66^. In addition, liver tissue samples were collected for subsequent molecular biology experiments (see the next section). Following analysis, the animals were fixed in 5% formaldehyde, preserved in 70% ethanol, and subsequently deposited in the fish collection of the Fish Genetics Laboratory, Faculty of Sciences, São Paulo State University, Bauru, São Paulo, Brazil, under the voucher numbers LG 13464, 13484, 17993 (BR), and LG 13310 (CN). All procedures were approved by the Ethics Committee on the Use of Animals at São Paulo State University (IBB/UNESP), under protocol 1204-CEUA/2019. The authors also complied with ARRIVE guidelines.

### DNA extraction and Short-reads sequencing data

Genomic DNA (gDNA) was extracted from two *C. modestus* specimens using the Wizard Genomic DNA Purification Kit (Promega), following the manufacturer’s instructions: one individual carrying three B chromosomes (3B), encompassing the two B variants described here, and one individual lacking B chromosomes (0B). Afterward, we checked DNA quality using 1% agarose gel electrophoresis. Subsequently, genomic DNA samples were sequenced on the BGISEQ-500 (2×100 bp PE) for both the 0B and the 3B individual. Sequencing generated 8,182,282 read pairs, corresponding to approximately 1.64 Gb, for the 3B individual and 6,121,279 reads pairs, corresponding to approximately 1.22 Gb, for the 0B individual. The estimated sequencing coverage was approximately 1,54× and 0,8× for the B-lacking and B-carrying individuals, respectively. Raw sequencing data quality was assessed using FastQC v v0.11.9^67^ make the data publicly available, we deposited short-read libraries in the Sequence Read Archive (SRA) of the National Center for Biotechnology Information (NCBI), under the accession numbers: SRR26178311 and SRR26178312.

### Bioinformatic protocols

Genomic libraries were quality-trimmed with Trimmomatic^68^, and reads were cropped to a uniform length of 100 bp to standardize the downstream analyses. The satellite DNA catalogs of each sample were independently characterized through successive iterations of TAREAN^69^, implemented within the RepeatExplorer platform and accessed through its public Galaxy server (https://repeatexplorer-elixir.cerit-sc.cz/galaxy). Four iterations were required to complete each catalog. In each iteration, a random subsample of 2 × 500,000 reads was generated using seqtk (https://github.com/lh3/seqtk) and analyzed with TAREAN. After each iteration, the forward and reverse read libraries were independently screened with DeconSeq^70^ against the satDNA sequences identified in the preceding iteration using a custom deconseq_run.py wrapper available at https://github.com/fjruizruano/ngs-protocols/blob/master/deconseq_run.py.

Following the initial characterization, tandemly repeated sequences corresponding to other repetitive elements, such as multigene families, were identified and excluded. The 0B and 3B catalogs were then merged into a unified catalog. Satellite DNA monomers were aligned using MUSCLE^71^, and redundant sequences showing 100% alignment coverage and ≥80% sequence identity were collapsed into a single representative sequence. Based on monomer sequence similarity, satDNAs were classified as belonging to the same variant (>95% similarity), different variants of the same satDNA family (80–95% similarity), or the same superfamily (50–80% similarity), following the criteria established by^72^.

The abundance and divergence of each satDNA in each library were estimated independently with RepeatMasker^73^ through the script (https://github.com/fjruizruano/ngs-protocols/blob/master/repeat_masker_run_big.py, accessed on 10 October 2022) with randomly selected 2 × 5,000,000 reads for each genomic library. Relative abundances of satDNAs in each library were obtained as the quotient of mapped reads by the number of nucleotides analyzed. Then, we arbitrarily chose the B-bearing individual genomic library as a reference to name the satDNAs in decreasing order of abundance, following the nomenclature proposed by^72^. Finally, we searched for satellite DNAs putatively clustered on the B chromosomes as the quotient of satDNA abundances in the B-carrying library relative to those in the B-lacking library.

Considering our *in silico* analyses and FISH results (see results section), three satDNAs were clustered on the B chromosomes (CmoSat01-192, CmoSat02-108, and CmoSat58-47). Of these, we selected CmoSat58-47 for an intra- and intergenomic diversity analysis to obtain a detailed and reliable score of haplotype abundances in both genomic libraries. We were restricted to this satDNA since its monomer is shorter than our longer read sizes. For this, we subsampled both libraries to 1,000,000 reads and aligned against a dimer of the satDNA using RepeatMasker. Then, we extracted the aligned reads, mapped them against this dimer with Bowtie2^74^, and isolated the central region corresponding to a single monomer. To avoid biases introduced by sequencing errors, we removed all the monomers that were represented once, using a custom Python script (https://github.com/fjruizruano/ngs-protocols/blob/master/cd_hit_filter_size.py). We then aligned the resulting dataset with MUSCLE^71^ and DNA diversity analyses were performed with DnaSP 6^75^. We also generated a minimum spanning tree (MST) based on the pairwise differences of monomer sequences and considering their relative abundances using PHYLOViZ 2.0 software^76^.

### Primer design and Polymerase Chain Reaction (PCR)

Primers were designed to generate FISH probes based on the following criteria: (i) the ten most abundant satellite DNAs suitable for primer design; (ii) all satDNAs exhibiting 3B/0B abundance ratios greater than 1.5; and (iii) satellite DNAs exhibiting 3B/0B abundance ratios below 0.5. SatDNAs with monomers shorter than 40 bp were excluded because the forward and reverse primers would substantially overlap, preventing the design of an appropriate amplicon. Low-abundance satDNA families ranked below CmoSat100-46 were also excluded.

Because CmoSat10-33 had a monomer length of only 33 bp, it was excluded, and the next most abundant satDNA suitable for primer design was selected. Thus, the abundance-based group comprised nine of the ten most abundant satDNAs and the next eligible family. In total, 22 primer pairs were designed and tested (Supplementary Table S2).

PCR amplification was performed with an initial denaturation at 95 °C for 5 min, followed by 30 cycles of denaturation at 95 °C for 20 s, annealing at 52–60 °C for 40 s, and extension at 72 °C for 30 s, with a final extension at 72 °C for 10 min. Of the 22 primer pairs tested, 15 produced specific and reproducible amplification products suitable for probe preparation. Primer pairs that failed to produce a clear product of the expected size or generated nonspecific amplification were not used for FISH. For the 18S rDNA, a pair of primers (18SF, 18SR) that amplify a 1400 bp segment of the 18S rRNA was used for probe generation^77^, using the *C. modestus* DNA as a template. PCR products were verified by electrophoresis on 2% agarose gels to confirm successful amplification and consistency and were subsequently quantified using a NanoDrop spectrophotometer (Thermo Fisher Scientific, Branchburg, NJ, USA).

### Fluorescence in situ Hybridization (FISH)

The probes derived from the satDNA’s PCRs were labeled with Atto550-dUTP (red), Atto488-dUTP (green), Atto425-dUTP (cyan), or Atto680-dUTP (yellow) by Nick-Translation (Jena Biosciences, Jena, Germany) and used for multi-color FISH experiments. All the aforementioned satDNAs (here named CmoSatDNAs) were hybridized in the chromosomal background of B-lacking (CN population), 1, 3, and 5B-carrying individuals (BR population). The hybridization mixes were composed of 100ng of each labeled satellite DNA plus 50% formamide, 2xSSC, 10% SDS, 10% dextran sulfate, and Denhardt’s buffer at pH 7.0 in a total volume of 20 µl, following high-stringency conditions^78^. Briefly, glass slides containing metaphase chromosomes were aged for 1h at 60 °C, following treatment at 37 °C for 5 min with 0.005% pepsin solution (99 µl H2O, 10µl HCl, and 2.5 µl of pepsin (20 mg/ml)). Chromosomes were denatured in 70% Formamide/2×SSC at 72°C for 3 min, while probes at 85 °C for 10 min, then cooled at 4 °C for 2 min before application. Hybridization occurred overnight in a moist chamber at 37 °C. Next, slides were washed for 5 min in 1×SSC at 65°C, and 4×SSC/Tween at room temperature, following a quick wash in 1×PBS for 1 min. The slides were dehydrated in an ethanol series (70%, 85%, and 100%), before the counterstaining of chromosomes with DAPI, mounted in Vectashield (Vector Laboratories, Burlingame, USA).

### Comparative Genomic Hybridization (CGH)

For the CGH assay, the genomes of a B-lacking (CN) and a 1B-carrying (BR) individual were compared to a 3B-carrying one (BR). For this purpose, all gDNAs were extracted as described above and labeled with fluorochromes emitting green (Atto488-dUTP), light blue (Atto425-dUTP), and red (Atto550-dUTP) fluorescence, respectively, with the Nick Translation mix kit (Jena Bioscience). The final probe mixture comprised 500 ng of derived gDNA of each individual and 10 µg of C_0_t-1 DNA obtained by DOP-PCR of each individual^79^. These probes were then co-hybridized against the chromosomal set of the 3B-carrying individual, following the protocol of ^79^.

### Microscopy and image processing

We examined a minimum of 20 metaphase spreads per individual to determine their diploid numbers (2n), karyotype structure, presence or absence of B chromosomes, and FISH results. The photos were taken with an Axioplan II microscope (Carl Zeiss, Jena, Germany) and processed with Image-Pro Plus 4.1 software (Media Cybernetics, Silver Spring, MD, USA). Based on their arm lengths, we classified the chromosomes into four types: metacentric (m), submetacentric (sm), subtelocentric (st) and acrocentric (a).

## Supplementary Information

Supplementary Information.

## Data Availability

The datasets generated during and/or analyzed during the current study are available from the corresponding author on reasonable request. The catalog of satellite DNAs was deposited on the GenBank with accession numbers OR604368-OR604483 and raw reads are available in Sequence Read Archive (SRA-NCBI) under accession numbers SRR26178311(B-lacking) and SRR26178312 (B-carrying).
