# Supplementary material for: Molecular divergence and genomic composition of B chromosomes in the fish Cyphocharax modestus (Characiformes, Curimatidae)
Source: Sci Rep. 2026 Sep 9;16:28198. doi: 10.1038/s41598-026-66448-7 (PMC13558729; doi:10.1038/s41598-026-66448-7)
Supplement: Supplementary file 1 [file 41598_2026_66448_MOESM1_ESM.docx]

**Supplementary Information****Figure S1:** Mitotic metaphase from *Cyphocharax modestus* lacking B chromosomes (0B) highlighting the chromosomal location of CmoSatDNAs. The satDNA family names are indicated on the right lower, in red (Atto550-labeled), green (Atto488-labeled), light blue (Atto425-labeled) or yellow (Atto680-labeled). According to the adopted satDNA nomenclature, the monomer length is indicated by the number following the hyphen in each satellite name (see Table S1). Scale bar =5 µm.

**Figure S2:** Mitotic metaphase from *Cyphocharax modestus* lacking B chromosomes (0B) highlighting the chromosomal location of CmoSatDNAs. The satDNA family names are indicated on the right lower, in red (Atto550-labeled), green (Atto488-labeled), or light blue (Atto425-labeled). Scale bar = 5µm.

**Figure S3:** Mitotic metaphase from *Cyphocharax modestus* carrying one B chromosome (1B) hybridized with CmoSat58 (red signal) and 18S rDNA (green). Arrows indicate the signals of CmoSat58, highlighting the B chromosome and one autosomal pair, that also carries the 18S rDNA. Scale bar = 5µm.

**Figure S4:** Mitotic metaphase from *Cyphocharax modestus* carrying five B chromosomes (5B) hybridized with both CmoSat58(red) and 18S rDNA (green). The satDNA family names are also indicated on the right lower corner. B chromosomes are indicated by arrow tips. **a)** DAPI counterstained chromosomes, **b)** Hybridization with CmoSat58 labelled with Atto550-dUTP (red), **c)** Hybridization with 18S rDNA probe labelled with Atto488-dUTP (green). **d)** overlapped image of both hybridized probes and DAPI chromosomes. Scale bar = 5µm.

**Table S1:** Main characteristics of 116 satDNAs found in *C. modestus*.

|  | **S.F** | | **satDNA family** | | **A+T (%)** | **Abundance 3B** | **Abundance 0B** | **Abundance 3B/0B** | **Divergence 0 B** | **Divergence 3 B** |
| --- | --- | --- | --- | --- | --- | --- | --- | --- | --- | --- |
| 5  1  6  1  5  7  4  6  8  7  3  2  3  10  10  8  6  10  10  10  10  8  9  7  4  10  10  10  10  9  10  9 | | CmoSat01-192  CmoSat02-108  CmoSat03-74  CmoSat04-187  CmoSat05-41  CmoSat06-317  CmoSat07-191  CmoSat08-41  CmoSat09-772  CmoSat10-33  CmoSat11-64  CmoSat12-20  CmoSat13-2520  CmoSat14-158  CmoSat15-65  CmoSat16-36  CmoSat17-42  CmoSat18-31  CmoSat19-647  CmoSat20-50  CmoSat21-34  CmoSat22-40  CmoSat23-1043  CmoSat24-41  CmoSat25-41  CmoSat26-30  CmoSat27-72  CmoSat28-43  CmoSat29-87  CmoSat30-52  CmoSat31-66  CmoSat32-39  CmoSat33-23  CmoSat34-130  CmoSat35-36  CmoSat36-182  CmoSat37-43  CmoSat38-991  CmoSat39-67  CmoSat40-31  CmoSat41-100  CmoSat42-21  CmoSat43-22  CmoSat44-30  CmoSat45-44  CmoSat46-48  CmoSat47-947  CmoSat48-61  CmoSat49-938  CmoSat50-1638  CmoSat51-308  CmoSat52-183  CmoSat53-56  CmoSat54-53  CmoSat55-49  CmoSat56-166  CmoSat57-245  CmoSat58-47  CmoSat59-853  CmoSat60-28  CmoSat61-38  CmoSat62-42  CmoSat63-32  CmoSat64-664  CmoSat65-695  CmoSat66-60  CmoSat67-140  CmoSat68-408  CmoSat69-213  CmoSat70-776  CmoSat71-68  CmoSat72-164  CmoSat73-36  CmoSat74-31  CmoSat75-71  CmoSat76-16  CmoSat77-748  CmoSat78-20  CmoSat79-843  CmoSat80-654  CmoSat81-240  CmoSat82-37  CmoSat83-195  CmoSat84-22  CmoSat85-48  CmoSat86-126  CmoSat87-1128  CmoSat88-172  CmoSat89-1371  CmoSat90-23  CmoSat91-20  CmoSat92-628  CmoSat93-48  CmoSat94-39  CmoSat95-48  CmoSat96-232  CmoSat97-126  CmoSat98-47  CmoSat99-37  CmoSat100-46  CmoSat101-694  CmoSat102-25  CmoSat103-39  CmoSat104-30  CmoSat105-20  CmoSat106-582  CmoSat107-31  CmoSat108-30  CmoSat109-228  CmoSat110-434  CmoSat111-18  CmoSat112-23  CmoSat113-350  CmoSat114-860  CmoSat115-319  CmoSat116-755 | | 64.1  52.8  52.7  64.7  65.9  57.7  59.2  61.0  58.9  72.7  53.1  60.0  62.7  49.4  64.6  58.3  57.1  51.6  52.9  40.0  58.8  52.5  41.8  48.8  68.3  46.7  62.5  53.5  56.3  55.8  60.6  53.8  73.9  50.0  69.4  50.5  30.2  62.1  61.2  54.8  56.0  71.4  59.1  56.7  52.3  52.1  65.3  54.1  51.5  53.1  53.6  57.4  53.6  58.5  59.2  51.2  51.8  44.7  59.2  57.1  55.3  64.3  65.6  55.0  51.8  56.7  47.9  57.6  54.0  54.5  63.2  58.5  41.7  54.8  60.6  50.0  56.8  55.0  52.7  48.5  38.3  56.8  51.8  50.0  50.0  55.6  56.1  48.8  53.1  56.5  55.0  53.7  56.3  56.4  47.9  41.8  54.0  40.4  45.9  58.7  48.3  56.0  51.3  53.3  65.0  61.2  41.9  56.7  52.2  52.3  44.4  52.2  38.6  52.7  52.7  57.6 | | 0.015  0.014  0.007  0.007  0.004  0.002  0.002  0.002  0.002  0.002  0.002  0.001  0.001  0.001  0.001  0.001  0.001  0.001  9.72E-04  9.26E-04  8.99E-04  8.77E-04  8.34E-04  6.84E-04  6.48E-04  6.29E-04  6.21E-04  5.92E-04  5.80E-04  5.11E-04  5.02E-04  5.00E-04  3.79E-04  3.79E-04  3.72E-04  3.72E-04  3.68E-04  3.67E-04  3.64E-04  3.59E-04  3.56E-04  3.51E-04  3.41E-04  3.38E-04  3.37E-04  2.76E-04  2.65E-04  2.63E-04  2.58E-04  2.50E-04  2.45E-04  2.43E-04  2.30E-04  2.28E-04  2.20E-04  2.08E-04  1.99E-04  1.90E-04  1.87E-04  1.78E-04  1.71E-04  1.67E-04  1.66E-04  1.64E-04  1.63E-04  1.55E-04  1.53E-04  1.47E-04  1.45E-04  1.45E-04  1.42E-04  1.36E-04  1.34E-04  1.33E-04  1.32E-04  1.31E-04  1.30E-04  1.29E-04  1.27E-04  1.25E-04  1.20E-04  1.16E-04  1.15E-04  1.14E-04  1.13E-04  1.11E-04  1.09E-04  1.07E-04  1.04E-04  1.03E-04  9.42E-05  9.39E-05  9.05E-05  9.01E-05  8.84E-05  8.33E-05  8.31E-05  8.31E-05  8.07E-05  7.84E-05  7.64E-05  7.46E-05  6.28E-05  5.79E-05  5.17E-05  5.04E-05  4.82E-05  4.42E-05  4.42E-05  4.19E-05  3.58E-05  3.35E-05  3.06E-05  2.96E-05  2.78E-05  2.49E-05 | 0.013  0.013  0.008  0.004  0.003  0.002  0.002  0.001  0.002  0.001  0.002  0.002  0.001  0.001  0.001  0.001  0.001  0.002  1.07E-03  1.05E-03  8.88E-04  1.54E-03  9.91E-04  7.82E-04  4.97E-04  8.49E-04  4.77E-04  7.08E-04  5.91E-04  4.82E-04  2.96E-04  6.02E-04  3.31E-04  6.90E-04  6.04E-04  1.74E-04  9.22E-04  3.21E-04  3.06E-04  5.45E-04  4.25E-04  3.14E-04  1.57E-04  3.77E-04  9.43E-05  2.93E-04  2.43E-04  2.68E-04  1.93E-04  1.73E-04  3.07E-04  4.40E-05  9.95E-05  3.28E-04  2.80E-04  5.64E-04  1.60E-04  5.56E-06  1.55E-04  2.33E-04  1.48E-04  2.21E-04  1.52E-04  1.44E-04  2.44E-04  1.36E-04  6.80E-05  1.21E-04  1.18E-04  1.11E-04  1.23E-04  1.39E-04  1.71E-04  2.73E-05  1.26E-04  1.93E-04  1.09E-04  3.15E-04  1.12E-04  1.30E-04  1.36E-04  1.22E-04  1.02E-04  2.21E-04  2.30E-04  9.04E-05  1.00E-04  8.34E-05  9.00E-05  1.17E-04  1.73E-04  7.02E-05  1.47E-04  8.28E-05  2.00E-04  9.40E-05  1.40E-04  1.83E-04  1.46E-04  6.76E-05  7.35E-05  2.50E-04  1.79E-04  1.43E-04  1.66E-04  5.93E-05  1.28E-04  1.11E-04  9.03E-05  3.96E-05  3.11E-04  1.04E-04  9.74E-05  8.79E-05  2.78E-05  1.60E-04 | 1.18  1.05  0.89  1.90  1.42  1.18  1.16  3.31  0.99  2.11  0.72  0.81  0.98  0.94  1.11  1.00  0.76  0.65  0.91  0.88  1.01  0.57  0.84  0.87  1.30  0.74  1.30  0.84  0.98  1.06  1.70  0.83  1.15  0.55  0.62  2.14  0.40  1.14  1.19  0.66  0.84  1.12  2.18  0.90  3.57  0.94  1.09  0.98  1.33  1.44  0.80  5.53  2.31  0.70  0.79  0.37  1.24  34.14  1.20  0.77  1.16  0.76  1.10  1.14  0.67  1.15  2.25  1.22  1.23  1.31  1.16  0.98  0.78  4.86  1.04  0.68  1.19  0.41  1.13  0.97  0.88  0.95  1.12  0.52  0.49  1.23  1.09  1.28  1.15  0.88  0.55  1.34  0.62  1.09  0.44  0.89  0.59  0.45  0.55  1.16  1.04  0.30  0.35  0.40  0.31  0.85  0.38  0.40  0.49  1.06  0.11  0.32  0.31  0.34  1.00  0.16 | 5.61  12.75  7.42  6.89  8.94  4.7  8.17  7.52  8.35  11.19  6.21  13.72  11.51  16.36  5.22  8.38  13.6  4.29  18.68  7.42  12.52  19.55  10.39  9.28  24.16  13.93  8.14  4.8  6.26  14.45  10.52  10.06  13.29  11.23  7.01  5.84  8.76  7.9  14.2  11.43  18.11  9.13  9.27  14.8  9.75  7.41  2.67  6.17  2.67  0.82  6.58  7.39  11.07  8.13  8.25  6.47  6.51  4.93  14.05  5.69  8.08  5.9  10.39  4.33  4.03  8.15  3.86  12.46  5.19  9.76  12.05  6.87  7.76  5.82  9.02  7.8  2.58  6.44  1.87  5.89  9.75  4.42  5.37  6.02  6.82  8.27  3.41  8.57  1.74  9.26  14.95  2.78  6.46  6.43  5.74  2.24  7.45  4.73  3.77  4.82  1.51  10.27  9.14  6.68  4.12  1.78  5.35  10.68  9.06  0.81  8  7.47  9.12  1.95  2.45  1.72 | 5.7  12.63  7.78  7.01  8.56  4.68  8.3  4.91  8.31  10.37  6.41  14.76  11.37  16.4  5.28  8.63  14.02  4.78  18.97  7.57  13.19  19.63  10.18  9.45  23.43  13.93  7.55  5.29  6.39  14.88  10.36  10.36  12.86  11.14  8.55  5.97  8.69  8.01  13.82  11.28  18.2  9.42  9.47  14.83  6.05  7.64  2.47  6.06  2.45  1.03  7.78  4.27  4.91  8.94  8.32  8.96  6.46  3.28  13.75  6.08  7.49  6.13  10.35  4.31  5.99  7.91  3.7  11.82  5.35  10.73  11.31  6.77  7.62  5.34  8.99  8.25  2.1  7.39  2.4  5.36  10.25  4.36  5.75  6.03  8.24  8.37  3.71  7.55  1.36  10.39  16.64  2.45  6.79  6.65  7.79  2.34  6.82  5.05  4.27  4.57  1.24  12.61  12.11  7.44  5.83  1.94  5.83  12.83  9.16  0.77  9.15  8.54  12.98  2.4  2.94  8.51 |

S.F: superfamily.

**Table S2:** CmoSatDNAs primers designed in the present study.

| **satDNA family** | **Primer F** | **Primer R** |
| --- | --- | --- |
| CmoSat01-192  CmoSat02-108  CmoSat03-74  CmoSat04-187  CmoSat05-41  CmoSat06-319  CmoSat07-191  CmoSat08-41  CmoSat09-772  CmoSat11-64  CmoSat31-66  CmoSat36-182  CmoSat37-43  CmoSat45-44  CmoSat52-183  CmoSat53-56  CmoSat56-166  CmoSat58-47  CmoSat67-140  CmoSat85-48  CmoSat95-48  CmoSat98-47 | GTTTTTGCAGGTTCCAGTTGTT  GGCGTCCTAGGGCATTACAA  CCACATAGCGCTACATAACAC  CCAAATATGTAAGAGCCATCC  CATTAACTGAGAACAATCGTGC  GCTTGTCTCTGTTGAAGGGC  GACCTAGGGTTAATGTTACAG  AGTATAGAACAGTACAGACT  TTCCTGTTGGGCGGAGCTAA  ACTGTAATGTGCCACCAGGT  CGCATTGTACACTTTCCCACT  GTCTACTACTTGTCTTCTACCA  TCAGAGCGCAGCAGTGTTCGC  GACACTGTACAGAACCGTTAC  TATTTCATGGGCCTGCTGACC  AGAGTCTTATAGCAGCAGGA  TTAGAGCTACGAATAGGTCTG  GTGGGTGATGTACAGGTC  GAGGTGAAGTGCGAGGGTCT  TTCAGACACACCCTCAGAC  CTACACACTGATCTCTACTAGC  TCACCACCAGACCAGAGTCA | TGCCTGTAGTTCAACCCCAA  CTGTTTCCCACGCTCTTAGA  CAGTGCTATATACTCCACCTG  GAACTCATCTCAAGCTTTTCAG  AGAACAATCGTGCTAACTCTGC  TCAGTGTCTCTCCTTGGCTT  TAACCATAACCCTAGCTGTAAC  CTCTCCTGAGGTAGTTATTA  GGTGGGAATGTAAAAGTCGTC  CATGCTATGTGACAGTGCTG  CCCTCTAGTGGACTTTATTTGA  CCTCCTTTAGAGCGGTGTG  GCCGAGGACGAGCGAGGGA  TGGTAGAGAGGTGAAGGAAC  TGTGGTTAAGCTGTGCCTATTA  ATAACCAGTCTCCATACTG  CCCTAACCCTAGCTCCAG  CACTGATCTACAGCCAGAGT  CACCACCACCTCCCTTAGT  GAGCAGTGTGCAGTGATTGG  TTGGCTGGGGTGGTACAGT  GGCTGTAATGAGGCGGTG |
